# The contributions of study abroad to home countries: an agential perspective

**DOI:** 10.1007/s10734-022-00980-z

**Published:** 2022-12-09

**Authors:** Yusuf Ikbal Oldac

**Affiliations:** Department of Education, University of Oxford, Oxford, UK

**Keywords:** International students, International higher education, Agency, Societal contributions, Turkey

## Abstract

Contributions to home country after international higher education (IHE) have long been considered within the traditional frameworks of brain drain or brain circulation. However, recent scholarship has hinted at more nuances into this issue than what has been predominantly discussed. This study focuses on IHE graduate agency to investigate the contributions of studying abroad to a home country. It builds from international-comparative fieldwork that included interviews with 50 recent Turkish IHE graduates who studied in four purposefully selected countries—Azerbaijan, Bulgaria, Germany and the UK—and who either stayed or returned to their home country afterwards. The findings highlight the role of agency in IHE graduates’ contributions to their home country. Returning to the home country does not equate to contributing to it, as some participants expressed that they contribute better from abroad while others refuse to contribute even after returning. The study also demonstrates that combining individual agency with push–pull factors emanating from the home country provides a more holistic explanation, as the home country dynamics have been found to be influential on agential stances regarding contributions.

## Introduction

International higher education (IHE) provides a significant opportunity for individuals to pursue quality education abroad. Through such experience, the individuals obtain a chance to develop new skills and knowledge, obtain new friendships and networks and form novel civic values (Knight, 1999). With increased capabilities newly formed through IHE, the assumption is that the graduates will have a higher potential to contribute to their home countries and the wider society.

Yet, the contributions of IHE graduates to their home countries have not received adequate attention in the IHE literature (Campbell, 2020; Tran & Vu, 2017). The existing scholarly literature has traditionally positioned the discussions on contributions to home country in IHE within the frames of brain drain, gain or circulation—which are terms used to refer to the migration of highly skilled individuals (Saxenian, 2005). The nomenclatures used to refer to this phenomenon in the literature vary, and studies use different nomenclatures to convey different meanings (e.g. Johnson & Regets, 1998; Teferra, 2005). These nomenclatures include brain drain (e.g. Commander et al., 2004; Meyer & Brown, 2003), brain mobility (e.g. Kenway & Fahey, 2011; Teferra, 2005) and brain circulation (e.g. Olang, 2014; Tung, 2008).

The studies that focus on this line of literature tend to argue that highly educated individuals emigrating from developing countries have been educated in government-subsidised educational institutions from early in their lives until their higher education (Marsh & Oyelere, 2018). Therefore, when these people emigrate, all the investments on the development of individuals are seen as losses for sending countries because the returns on the investment are reaped by another country and future tax revenue is lost (Marsh & Oyelere, 2018). Although remittances sent back by the migrant IHE graduates mitigate the monetary losses of the sending country, according to Marsh and Oyelere (2018), these remittances are no match for the potentially lost future tax revenues and fiscal expenses made on subsidising their education. In the brain drain/gain/circulation literature, the question mostly revolves around whether individuals who went abroad to obtain a degree are a lost skilled workforce if they do not return and the subsequent consequences of this situation on their contributions to their home countries (e.g. Gribble, 2008; Hart, 2006; Séguin et al., 2006).

However, this study does not situate the contributions of IHE graduates to society within the brain drain, gain or circulation paradigms. These popular models, especially the idea of brain drain, may have some problematic assumptions. These assumptions include internationally mobile students being able to contribute to their home country by returning after completing their degrees abroad (cf. Campbell, 2017), sending countries losing their skilled workforce when these students do not return and each person’s identity belonging to only one nation and them showing loyalty to that nation (Rizvi, 2005b). Rizvi (2005b) contended that brain drain is a term that was coined in the 1960s by newly formed small states that worried about the loss of their skilled workforce to more developed countries and that it is becoming increasingly problematic in today’s world. Brain drain essentially rests on the classical understanding of the relationship between social identities and the nation-state, which some authors think may be weakening in the globalising world (Bauman, 2000; Giddens, 1991). Some criticism of the brain drain paradigm has emerged within the paradigm itself. Tung (2008) argued that people used to have a more clear-cut understanding of who constituted expatriates are, who constituted host country nationals are, and how brain gain or brain drain are defined. These notions have become much more blurred in the modern world (Tung, 2008).

In addition, diaspora literature can also be relevant to the discussion of IHE graduate contributions to the home country. Diaspora groups and their contributions to the home country have been well examined in the literature (e.g. Beine et al., 2011; Taslakian et al., 2022). However, the studies that focus on the contributions of diaspora do not focus on IHE graduates specifically, although the groups on which they focus may include migrant graduates as part of the larger diaspora group (Cai, 2012; Welch & Hao, 2013, 2016). Meanwhile, those limited number of studies that do focus and differentiate IHE graduates within a diaspora group may not necessarily concentrate on the their specific contributions to the home country (Kim & Bamberger, 2021; Marini & Yang, 2021). ‘Diaspora’ in higher education studies is usually undertheorised and fragmented (Bamberger, 2021). Similarly, IHE does not receive the attention it deserves from diaspora studies (Oldac & Fancourt, 2021).

In contrast to the literature discussed above, a recently emerging line of scholarly work in the IHE literature has started investigating IHE graduate contributions to the home country specifically and without building on the discussed binary brain drain logic of whether studying abroad is a lost skilled workforce upon not returning (e.g. Campbell, 2019, 2020; Perna et al., 2015a, 2015b; Tran & Vu, 2017). This recently emerging line of literature indicates that IHE contributes to the students’ home countries in several ways, including having an impact on national policy (Campbell, 2017), improving bilateral diplomacy (Kent, 2018), playing a positive role in the internationalisation of higher education (Zahler & Menino, 2018) and building capacity at organisations in the home country (e.g. Kallick & Brown Murga, 2018; Perna et al., 2015a, 2015b). The following section discusses the main narratives in this emerging line of research.

### Main narratives in the literature for contributions to home country in IHE

The existing scholarly empirical work has built on three distinct narratives when researching IHE graduate contributions to home country (Campbell, 2018). The first of these narratives, which is also the most prevalent one, frames IHE graduate contributions using human capital theory (e.g. Campbell, 2016, 2017, 2020; Perna et al., 2015a, 2015b; Perna et al., 2015a, 2015b). For example, Campbell (2016, 2017, 2020), who has been recurrently publishing on this topic, has built on McMahon’s (1999, 2009) expanded theory of human capital to argue that students who studied abroad obtain new embedded qualities, which will help them contribute to their communities at home. According to McMahon’s extended human capital perspective (1999, 2009), human capital development is not just about increased private benefits such as income for graduates but also a potential contribution to society. He argues that these highly educated international education graduates would have a social life outside their work environment to which they can contribute by establishing better relationships with their community. In addition, Perna and colleagues’ works (Perna et al., 2014, 2015a, 2015b) are also among the studies that have expanded the human capital narrative. They have built on an earlier influential conceptualisation of human capital theory (Becker, 1993) in building their narratives in the papers.

Another narrative in contributions to home country literature is related to the human rights approach (Campbell, 2018). This narrative comprises studies that situate international education as part of one’s right to obtain quality education (United Nations, 1948). According to this approach, as quality education is one’s right, contributions to one’s home country afterwards, although desired, cannot be made compulsory. This narrative is especially valid for studying the expectations of scholarship providers. Some studies have looked into the contributions of internationally educated individuals with this perspective (e.g. Lehr, 2008). However, this approach has not been as popular as the human capital approach, especially in the designing of IHE scholarships for ensuring contributions to the home country after graduation (Campbell, 2018).

A third narrative to frame international student contributions is that of the human capabilities approach (Sen, 2000). This approach frames the goal of education as a process of increasing one’s choices and freedom and highlights an individual’s increased choices and agency after graduation. While the capabilities approach has been influential in sociological and human development studies, few studies have included this perspective in framing contributions of international students to their home countries (Campbell, 2018). In contrast, Sen’s emphasis on choices and individual agency (Sen, 1985) can play an important role in illuminating the discussions on societal contributions of IHE graduates, especially for situations in which graduates are reluctant or even against contributing to their home country. The existing scholarly work supports this notion by pointing out that returning to the home country does not automatically equate to contributing to it (e.g. Campbell, 2017) and not returning does not automatically equate to not contributing to it (e.g. Akçapar, 2009). The role of agency makes the difference here, which has been understudied in contributions literature (Tran & Vu, 2017).

In this burgeoning line of research, a few influential publications have drawn attention to the gap on the focus on individual agency in investigating IHE graduate contributions (Campbell, 2018; Tran & Vu, 2017). The current study contributes to this literature by filling out this gap building from new empirical insights from Turkish students. The following section will delineate on the framework of this study, and the section afterwards will provide a background context for Turkish international students.

### Theoretical framework: an agential lens

In her chapter, Campbell (2018) provided a discussion of the notion of agency in international scholarship graduate contributions. She defines individual agency as ‘the ownership for decisions and actions made by a scholarship grantee, given the options available at the time. It is how an individual exercises their choices and weighs their interests and desires against a given range of possibilities and specific life goals (p. 172). In this study, I build on Campbell’s agential approach to IHE graduate contributions and delineate agential perspective to contributions to home country in IHE utilising Sen’s (1985) definition for individual agency. IHE graduates have freedom to attain whatever they decide to contribute to their home country and beyond it, as responsible agents.

This study’s agential perspective incorporates the push–pull model in its theoretical framework, building on Campbell’s (2018) proposition that individual agency goes hand in hand with push–pull factors (Li & Bray, 2007; Mazzarol & Soutar, 2002). The larger sociological discussions always pair any discussion of agency of individuals with the external factors, usually framed as the structure (Archer, 2003; Giddens, 1991). Agency is crucial in human actions, but it alone does not explain the whole picture and external factors needs to be incorporated. Push–pull factors are incorporated in the current work to discuss the external factors related to IHE graduate agency.

Push and pull factors are widely used in migration and international student mobility studies (Kondakci, 2011; Li & Bray, 2007; Mazzarol & Soutar, 2002). However, its use in framing IHE graduate agential contributions is relatively new. In this model, push factors are those that drive students away from their home, demotivating them from contributing to it. Meanwhile, pull factors are those that attract international students to specific destination countries. The latter factors may result in decreased motivation to contribute to the home country as well but not necessarily, as the graduates may still choose to contribute through different means such as creating diaspora connections or sending back remittances and investments, as will be discussed in the Findings section.

However, push factors may be stronger in affecting IHE graduates’ agential rejection or shifting allegiance to contribute to their home countries. Although the participants of this study have studied in diverse destination countries, they are all from Turkey. As such, they all share similar push factors, having moved from the same country for their international studies. Specifically in the Turkish context, push factors have emerged to be highly palpable and clear in the interviews, thus influencing how IHE graduates negotiate their agential decisions for contributing to their home country, the main topic of enquiry in this research.

### Turkish context and Turkish international students

International students are individuals who have relocated from one country to another to pursue an undergraduate or postgraduate degree education (OECD, 2017). When defined this way, close to 50,000 Turkish students were studying abroad in 2018, according to the most recent available data (UNESCO Institute of Statistics, 2022). However, Turkish international students have been understudied in both Turkish and the broader literature despite their large number.

Turkish IHE graduates show similarities and differences from other international students. They are similar in that they all go through the same dynamics of IHE: they all cross country borders to obtain a degree education, leave behind their existing networks and friends and form new ones, and witness and experience novel communal values in a new setting. In this respect, the selected group may be viewed as instrumental in gaining a better understanding of the IHE graduates’ overall perceived contributions (Ragin, 1992; Stake, 1996).

However, Turkish IHE graduates may differ from other IHE graduates in certain ways. One difference is that they are from an upper-middle-income country (World Bank, 2020) that is geographically near many high-income countries. As a result, they may have both the motivation and the means to cross borders (Beine et al., 2008). This circumstance makes Turkey susceptible to high levels of international student mobility and graduate migration, a topic of great significance in the societal contributions literature.

Turkey has also been undergoing a complicated and volatile political situation lately, notably the recent coup attempt in 2016. This volatile atmosphere has been noted by several international watchdogs, such as The Economist Intelligence Unit (2020) and Freedom House (2020), which argued that the freedoms in Turkey are declining. In addition to the political volatility, the last seven years leading up to 2020 has been marked with significant decreases in economic purchasing power in current US$ terms (World Bank, 2022). Such developments have created strong push factors, which are especially marked with the large-scale migration of highly educated white-collar professionals to other countries, as supported by Europe’s immigration and asylum statistics (Eurostat, 2022). These developments have created a rather pessimistic perspective for young adults studying abroad. In this sense, Turkish IHE graduates have intrinsic value, as Stake (1996) puts it, for studying IHE graduate contributions.

Having said these, this study does not consider Turkish students as a homogeneous group. The available datasets, such as UNESCO or World Bank, for selecting international students are based on nation-state categorisations. In this regard, Turkish students have been selected because the available datasets make it feasible to draw from such a categorisation.

## Methodological approach

This study was conceived as a qualitative study that drew its data from semi-structured interviews. In this section, I delineate the details of the methodological approach taken for this study.

### Participant selection

The participants of this study were 50 Turkish recent IHE graduates who studied in various host countries. Azerbaijan, Bulgaria, Germany and the UK were the four purposefully chosen host countries to go beyond one host country context and get an overall picture of agential contributions. Graduates who stayed in their host countries after graduation were interviewed in these countries, and returnee graduates were interviewed in Turkey. These countries were selected among the top 10 most popular destinations for Turkish students based on the UNESCO Institute of Statistics (2018) to ensure variety while preserving feasibility. The countries were chosen on the basis of factors such as historical/cultural links, political economy and the quality of higher education institutions. The inclusion of multiple country contexts was not to compare the findings between each country context one by one but to provide a holistic picture of the agential aspect of contributions to the home country after IHE. As the findings will indicate, the agential aspect goes beyond one host country context.

In each country, the participants were graduates of top-ranked universities. To achieve this goal, I used rankings such as Times Higher Education, QS World University Rankings and uniRank. Top-ranked universities were selected to account for the differences in quality and future opportunities offered by different types of higher education institutions. The options accessible after graduation might impact IHE graduates’ perceived contributions. Table 1 below shows participant distribution for each country and the universities from which they received their degrees.

**Table 1:** List of universities the participants obtained their degrees from with regard to country locations

Country of study	Number of participants	Interviewed in host/home country	Participant universities
Azerbaijan	12	5/7	Azerbaijan State University of Economics Baku State University Azerbaijan Technical University Baku Engineering University (Old name: Qafkaz University)
Bulgaria	12	5/7	Sofia University “St. Kliment Ohridski” University of National and World Economy Technical University – Sofia Medical University-Sofia
Germany	14	8/6	Humboldt University Free University of Berlin Ludwig Maximilian University of Munich Technical University of Munich
UK	12	6/6	University of Oxford University of Cambridge University College London Imperial College London London School of Economics University of the Arts London

I screened the participants using a combination of snowballing and LinkedIn’s search tool. The participants had a balanced distribution of return status: migrant graduate = 26, returnee graduate = 24. Migrant graduate means IHE graduates who were residing in their host countries during the time of the interview, whereas returnee graduate means those who had returned to Turkey at the time of the interview. The participants also had a balanced gender distribution (women = 20, men = 30) congruent with the total international student population. Moreover, they graduated from a broad range of study areas, ranging from social sciences to engineering and sciences. The purpose of the study was to include degree-level graduates to gauge the perspectives of those who stayed longer in their study abroad experience, as compared to short-term mobile individuals (e.g. semester-long visits). The participants of this study eventually constituted of undergraduate and master’s graduates (respectively: 6/6 Bulgaria, 12/0 Azerbaijan, 4/10 Germany and 2/10 UK).

The participants were all young adults, meaning their age ranged from 20 to 35 years old (Armstrong, 2007), to obtain insights from recent international student mobility experiences and to ensure that the participant responses were comparable. In addition, the study did not include Turkish government scholarship recipients. Turkish government-funded students have to work a certain amount of time in a pre-specified organisation in Turkey after graduation, which can significantly influence graduate perceptions on their societal contributions. Moreover, the majority of studies focusing on contributions to home country focus on scholarship recipients, thus signalling an incentive to study non-scholarship recipients.

### Data collection and analysis

I adhered to the British Education Research Association’s (BERA) (2018) research code and received clearance from The University of Oxford’s research ethics committee. I acquired participants’ informed consent and anonymised their data.

All of the interviews took place between September and March 2020. I conducted the majority of the interviews in person. The interview durations varied from 50 to 75 min. Only four of the 50 interviews were performed through Skype. The interviews were conducted in Turkish because it is the participants’ and the author’s native language. The data from all of the interviews were transcribed. The full coverage of interviews focused on the larger topics of self-formation and societal contributions in IHE, as this study is part of a larger doctoral thesis (Oldac, 2021). Self-formation is a relatively new framework that positions students as agential self-educating individuals and focuses on their holistic formation (Marginson, 2014). This particular research focuses on the findings related to the contributions to home country after IHE.

I used thematic analysis to analyse the data, as described by Miles et al. (2019). The analysis was carried out in Turkish because the data were in that language. However, the selected portions were subsequently translated into English for publication. This examination involved inductive coding in relation to the emergent patterns. As indicated above, the underlying study was not directly focused on the impact of push factors emanating from the home country, but they emerged as a strong pattern for the participants of this study. Hence, a fresh thematic analysis of existing qualitative data was conducted. Both frameworks (i.e. agential self-forming individuals and push factors from the home country) emerged to be viable and coherent in framing contributions to the home country during the data analysis. The more refined themes that emerged from the re-analysis are discussed in the following Findings section.

## Findings

This section reports the interview data under three emergent themes, namely, agential rejection to contribute, ‘better from abroad’ and shifting allegiances and transnationality. These themes emerged without a specific prompt about unfavourable push factors emanating from Turkey, as indicated earlier. Thus, a deeper exploration on these themes has become important.

### Agential rejection to contribute

One of the main themes that emerged from the interviews was the agential decision of rejecting to contribute to the home country. As discussed earlier in the theoretical framework part, the excerpts below support an agential decision to reject contributing to home country, Turkey, citing some of the perceived factors pushing them away from doing so. The participants who explained they do not want to contribute to home country dwell on negotiations in their minds whether their home country, Turkey, deserves their efforts in this matter. Simge’s quotation below exemplifies this perspective:

Should I provide this support to Turkey? Do I want Turkey to develop? … Does Turkey deserve it? Or did it give me something to deserve it in terms of education and social rights? … I am not sure about that, and I guess it doesn’t deserve it. *Simge, Bulgaria, Returnee graduate*

Notice how she questioned whether her own country has given her anything at all. When considering that primary, secondary and tertiary education provided by state institutions in Turkey are freely provided, such an argument may not be entirely true. However, according to the reflections shared under this theme, the situation is not that simple.

The participants often indicated their good intentions towards their home countries. They explained that they are positively inclined towards contributing to their home country. However, the interviews also unearthed a negative tone and a despair that they do not see a reciprocity for their good intentions. Defne, for example, explains below the uneasy situation she faces when it comes to contributing to her home country.

I would be happy to contribute to Turkey, I mean our beautiful country, but on the other hand, I do not see reciprocity for this. I have such positive feelings towards Turkey, but Turkey does not seem to have such positive feelings towards me. *Defne, Germany, Migrant graduate*

Not seeing any reciprocity regarding ‘such positive feelings’ is relatable to the push factors discussed in the theoretical framework of the study. International education graduates are pushed further away from contributing to their home countries, as they perceive that no one appreciates it. The participant interviews included abundance of explanations such as Defne’s above. Some participants even went beyond and explained that contributing to home country can even be harmful to them. Kemal’s interview excerpt below illustrates this point:

The contribution I can give is mostly in the field of sustainable energy. I want to do this, but Turkey does not want to get it, and you wear out after a while. You question: what am I working for? I mean it will not respect you. It will denigrate you. It will not care about you. People don’t accept your lifestyle. . . . I used to say I should contribute to my country but not that much anymore. *Kemal, UK, returnee graduate*

Notice the strong wordings such as ‘denigrate’. Kemal feels that contributing to Turkey, his home country, after graduation is not just pointless but also a tiring effort that makes him feel burnout. Hence, he does not feel like contributing to his home country anymore.

Political, economic, social and cultural factors pushing people from Turkey need further attention in understanding IHE graduates’ agential rejection to contribute to their home country. Participants repeatedly mentioned governmental policies and practices as an important reason behind their *Agential Rejection.* To illustrate, Salih provided specific reasons for why he has never thought of contributing to his home country before:

It will seem a little selfish, but I have not thought of contributing to the country. So why should I help this government? There may be something like why I should help the country that voted for this government. Maybe this is why I didn’t think about it. *Salih, UK, migrant graduate*

As can be seen, Salih equated contributing to his home country to contributing to the incumbent government, which seems to demotivate him to do so. Salih’s explanation in this quotation seems to be connected with others in this theme.

In addition, the participants also highlighted that they do not have any legal obligation to return and contribute to their home country either. They were not government scholars. Onur’s reflection below illustrates this situation well:

Why don’t you come back and contribute after studying abroad? Well, it’s our own life, our own choice. Maybe because of our resentment, maybe we saw some negativities in Turkey and not here, and so we stay. We didn’t study here with a government scholarship. We paid with the financial support given by our own family or the income we earned by working while studying. *Onur, Bulgaria, migrant graduate*

The role of agency is crucial in contributions to home country after studying abroad, and it is even more highlighted when IHE graduates are not bound by any legal requirements. The interview data shared in this section demonstrate how IHE graduates can agentially reject to contribute to their home countries regardless of their country of study and return status.

### ‘Better from Abroad’

This theme specifically highlights the perspectives of those participants who stayed in their host country after graduation but still dwelled on their intention to contribute to their home country. This theme can be understood better with state and nation separation discussed by Kim and Bamberger (2021). IHE graduates still want to contribute, but this intention is due to their allegiance to the nation, separating it from the current governing state. The participants argued that their contributions to their home country are/would be ‘better from abroad’. They mostly cited two push factors: that no adequate opportunities are available in Turkey (such as the necessary labs for research or large corporations focusing on their field) and/or because their jobs are more difficult to do in Turkey due to the restrictive atmosphere (e.g. academic atmosphere). Quotation marks used for the title of this theme denote that this term is an in vivo code. Below, sample excerpts are shared to provide more details about this theme.

To start, Yasar is a finance graduate who currently resides in London, UK. During his interview, he discussed extensively that his home country lacks big investment banks such as the one where he works. In the excerpt below, he explained how his contribution could be much better and on a bigger scale if he stayed in London instead of returning:

I think if I return, I won’t contribute as much as I do from here. … My current company doesn’t have any investment in Turkey yet. … Even if investing [in Turkey] becomes easier, somebody needs to tell potential sponsors about it. … 14 billion dollars a year enters [to Turkey in our area]. … Our average investment at once is a 1 billion turnover. If I do one project on such a scale in Turkey, that 14 billion will become 15 billion. *Yasar, UK, migrant graduate*

For the explained reason, Yasar has taken the agential decision to stay in the host society where he obtained his international degree. Later in his interview, Yasar also discussed the increasingly not-so-attractive restrictive atmosphere in his home country. Other participants were also conscious of the increasingly restrictive atmosphere in Turkey. This circumstance is also relevant for those who are interested in conducting academic studies that may include critical perspectives towards contemporary issues in Turkey. One example is presented in the excerpt below from Zeynep. She repeatedly mentioned the restrictive atmosphere for studying certain topics in Turkey. Building on such claims, she explained that she can conduct such studies in Germany freely and hence contribute to her home country from abroad:

Turkey, Italy and Greece—these have vast numbers of unemployed women, and some of them are not even considered unemployed … For example, this is one of the topics I want to work on. … I can work here on this population of women, who are not even considered unemployed in Turkey. I do not have to return to Turkey to do it. *Zeynep, Germany, migrant graduate*

As the sample excerpts indicate, push factors such as restrictive atmosphere in the home country lead the participants not to return home. Nevertheless, they still would like to contribute to it from abroad.

Contributing better from abroad may come in different forms and not just limited to conducting critical academic studies. Ayten is another participant who is an international graduate of comparative politics. In her interview, she explained that she is advising one of the parliament members of the main opposition party in Turkey.

A: My doctorate is in politics, and I look at the oppositions in Venezuela and Turkey … so that it is comparative. I’m looking at the mistakes of the oppositions. Unfortunately, the strategies, the style, they are similar.

Researcher: Your counselling will help a lot, then. Maybe you can show a way to [the main opposition party in Turkey].

A: Yes, yes. … [and this would work better if they are more] open to dialogue and self-criticism. *Ayten, UK, migrant graduate*

As the excerpt indicates, Ayten is putting her newly acquired information and skills into use to contribute to her home country, but she does that from abroad. Moreover, she explained later in her interview that she is even working in low-paying jobs in the UK to not to return to her home country, citing the earlier discussed push factors.

Another form of contributing from abroad could be doing business with and importing products from Turkey while staying in the host country. These people mostly discussed their reluctance to return to their home country after their education and argued that their contribution would be much bigger if they stayed. Berke’s example below illustrates this perspective:

I sell Turkish brands. Let’s say X million Turkish liras a year so that you can understand the scale of it thanks to the exchange rate. We contribute to Turkey that much. *Berke, Azerbaijan, migrant graduate*

Overall, the participants of whom I shared their reflections in this section dwelled on their goodwill to contribute to the nation ‘better from abroad’. The general thinking among them was that IHE graduates who have not returned to their home countries will continue to grow as ‘the escape from Turkey will continue in the future due to the political and economic situation in Turkey’ (*Ahmet, Germany, migrant graduate)*, which indicates that the theme of ‘better from abroad’ will gain even more importance*.*

### Shifting allegiances and transnationality

This theme gathers together the excerpts that indicate how the participants negotiated their transnational identity shifts, similar to those discussed by Rizvi and colleagues (Dolby & Rizvi, 2008; Rizvi, 2005b). According to Rizvi (2005b), such transnational transformations could occur during any international study experience; however, the interview data of this study indicated that the push factors emanating from Turkey are facilitating and even accelerating international students’ allegiance shifts. To illustrate, for Zeliha below, belonging is not to a specific country or bordered land anymore; it is rather to the ‘ideals’ she created:

For me, there is no direct belonging to Turkey, but to the ideals I created. This is not like any country or national unity border. If it is a country compatible with my ideals, I would gladly return, live there and work even for little money. *Zeliha, UK, migrant graduate*

Zeliha above highlights her transnationality: she does not feel she belongs to any country anymore. Her allegiance is now to her newly formed ideals and whichever country is compatible with them. Zeliha is not alone in this thinking. The excerpt below from Rana’s interview also signifies this perspective:

I am from here (Turkey), but it is questionable whether I feel I belong here… because my heart is broken in general. I saw that you could live more pleasantly. . . . So, I want to create added value, but will this be country-based? No, it will be human-centred. I think people are global and the world is a global place. *Rana, Azerbaijan, returnee graduate*

Notice how she associates her overall disappointment with Turkey to her feeling of belongingness. She explains that she will make agential efforts to contribute to humanity instead rather than to her home country specifically, even though she has already returned from studying abroad. Shifting allegiance and transnationality is very visible.

Furthermore, the acceleration of transnationality and shifting allegiances is visible even for those who already felt they were world citizens before their international higher education experience. The excerpt below from Aysel illustrates this notion:

As a person who sees herself a bit more like a world citizen, I think that the issues of the world concern me. Therefore, I choose my work accordingly. … I was never like a person who thought that I should advance my country from one point to another, but studying abroad further lowered my perceptions that I am attached to one nation. *Aysel, UK, returnee graduate*

The interview data shared in this section indicate that IHE graduate contributions are impacted by the push factors in home country strongly. These push factors impact IHE graduates’ ‘perceptions in a way that [they] feel less attached to one nation’ (*Aysel, UK, returnee graduate*). As a result, their allegiance shifts from their home country.

## Conclusions and discussion

This study examined how international study graduates negotiate their agential decisions on contributing to their home country, Turkey, at a time when push factors emanating from home country is highly palpable. The findings section specified three emergent themes in relation to the earlier discussed framework of the paper: agential rejection to contribute, ‘better from abroad’ and shifting allegiances and transnationality. All three themes point out the importance of individual agency in IHE graduate contributions, which has been mostly neglected in this line of research, echoing Tran and Vu (2017) and Campbell (2018).

Agency is an important concept mostly missing in the brain drain or gain literatures. Return status has been mostly seen as the defining factor for deciding whether an international graduate is a ‘lost workforce’ (cf. Campbell, 2020; Dassin, 2009; Marsh & Oyelere, 2018). The analysis in this study indicates that this notion is not necessarily true, as some participants argued that they would do everything in their hands not to contribute to their home countries even though they had already returned their home country. Meanwhile, others explained that their contributions would be better if they did not return to Turkey, thus supporting some of the studies that problematise return status in this line of research (e.g. Campbell, 2019; Rizvi, 2005a; Tung, 2008). Return status may still have an impact on the nature of the graduate contributions and their overall perceptions of contributions to their home country; however, individual agency is key here. The findings indicated that agency has a larger role in IHE graduate contributions than the scholarly literature on this topic has acknowledged so far (Tran & Vu, 2017).

Moreover, as discussed earlier in the theoretical framework, the larger sociological discussions of human agency always pair agency with external factors, often termed as the structure (Archer, 2003; Giddens, 1991). Human agency and external factors are always in interaction with each other. Hence, I followed Campbell’s suggestion in this study and included push–pull factors to incorporate the external factors in understanding the agential decisions of IHE graduates. The findings revealed that combining individual agency with push–pull factors provides a more holistic explanation of contributions to the home country. This study specifically focussed more on the push factors, as they emerged to be highly prominent in the participants’ perceptions of their contributions to Turkey. As the analysis illustrated, a significant number of participants (theme 1) demonstrated an agential stance to reject contributing to their home countries, (theme 2) argued that they would contribute better from abroad due to their commitment to the nation but not necessarily to the current governing state, echoing the distinction made by Kim and Bamberger (2021), and (theme 3) problematised their sense of allegiance to a bordered area called home country (Dolby & Rizvi, 2008; Rizvi, 2005b).

Based on these findings, the recent developments in Turkey, especially the alienating impact of governmental policy and practices, have been ‘pushing’ away its own citizens who have studied abroad from contributing to their home country. Many participants explicitly discussed this circumstance in their interviews, as some of these discussions are included in the Findings section. Participants had a shared thinking that Turkey is increasingly becoming more restricted, which is supported by several other sources (Economist Intelligence Unit, 2020; Freedom House, 2020), and economically less attractive, as measured by per capita purchasing power (World Bank, 2022). Considering that IHE graduates are highly educated and self-formed young adults (Marginson, 2014, 2018; Marginson & Sawir, 2012) who have a long, productive time ahead, it is in Turkey’s best interest to win their hearts back. The latter will be discussed more under the following policy implications section.

Transnationality and problematising the sense of belonging to one’s home country have also emerged as a strong theme from the analysis. Transnationality in international education is well researched and discussed (Bamberger, 2019a; Rizvi, 2005a, 2005b). However, the analysis here indicated that push factors emanating from home have facilitated and even accelerated the participants’ shift in allegiance and transnational negotiations in their mind. Similar to Rizvi’s counter arguments towards brain drain literature (Rizvi, 2005b), the contributions to home country discussions could also be considered to rest on the idea of the relationship between social identities and the nation-state, even when they are framed differently than most brain drain studies. However, one main point here is that even though international education graduates who are motivated to contribute to overall humanity are desirable, those graduates who specifically want to exclude their home country from this equation are a significant area of research.

### Policy implications

In this section, policy implications are discussed. Although these implications will be discussed with the specific example of Turkey, they are also relevant to other upper-middle-income country contexts that are undergoing politically volatile situations. As indicated earlier, one in three participants repeatedly argued that they do not feel included in Turkey and that their contribution is not wanted. It is in Turkey's best interests to win back the hearts of these highly educated young adults who have a long and productive future ahead of them. The first and most important step to do so is to create a more inclusive policy atmosphere that sends out positive messages. International education graduates would not want to contribute to an atmosphere in which they feel they are ‘not accepted, respected and even denigrated’ as Kemal, Defne and others argued.

Notably, the participants of this study were not Turkish government scholarship recipients, as explained in the Participant selection section. Turkish government-funded students have to work a certain amount of time in a pre-specified organisation in Turkey after graduation. Such mandatory measures do not apply to this study’s participants, as they are not officially obliged in such ways. Thus, the focus should be on soft approaches.

In this regard, Turkey could focus on creating promising opportunities at home and providing a welcoming environment to maximise the contributions from internationally educated Turkish individuals, similar to what China, the largest sending country, has been doing (Pan, 2011). In addition, Israel could be a good example, as it used its diaspora to boost the internationalisation of its higher education (Bamberger, 2019b). Israel’s case indicates that, when treated well, diasporas can help universities receive more international students, which is a positive contribution to the development of universities.

In addition, Turkish embassies abroad could make positive gestures, even small ones, to help these young adults feel that they are not alone in their journey abroad and that their home country is there for them. Sending out small gifts, such as calendars on important national days, or reaching out during difficult times, such as the current COVID-19 pandemic, though small gestures, can be instrumental in winning the hearts of these people back. To illustrate, the Chinese embassy in the UK has been sending small gifts and cards to show support for its nationals.

In addition to Turkish young adults who have studied abroad, over 6.5 million Turks are residing outside Turkey (Republic of Turkey Ministry of Foreign Affairs, 2020). This number is significant by any criteria. Although not all of these Turks residing outside of Turkey are as highly educated as IHE graduates, they can still contribute to Turkey in certain ways. These groups need to be engaged as diaspora communities to ensure their contribution to Turkey. However, this issue has not been studied adequately in the literature. The recommendations provided above could help engage the overall diaspora of Turks abroad, but we need more studies that incorporate the specific dynamics of Turkey and Turkish people (Oldac & Fancourt, 2021).

### Limitations

The study utilised interviewing method, which is a self-reported method and subject to self-desirability issues. That is, the participants’ responses may have been influenced by what they thought was more socially desirable, especially given that the questions were on contributions to home country. This approach may have led to over-emphasising socially acceptable and under-emphasising socially bad behaviours. Several measures were taken to alleviate this potential issue. One of them was to use a semi-structured interview guide. Semi-structured interview guides helped structure the questions and ensure more relevant responses (Patton, 2015). Moreover, during the interviews, probing questions were asked, and examples were requested for claims made by participants.

Lastly, the participants were from Turkey. The agential lens discussed in this study is likely relevant to students from different backgrounds as well. However, the push and pull factors would be different. As discussed in the context part, while some findings bear instrumental value in understanding overall international students, some bear intrinsic value specific to Turkish students. The conclusions and discussions should be interpreted with these considerations in mind.
